# Insights into Clinical, Genetic, and Pathological Aspects of Hereditary Spastic Paraplegias: A Comprehensive Overview

**DOI:** 10.3389/fmolb.2021.690899

**Published:** 2021-11-26

**Authors:** Liena E. O. Elsayed, Isra Zuhair Eltazi, Ammar E. Ahmed, Giovanni Stevanin

**Affiliations:** ^1^ Department of Basic Sciences, College of Medicine, Princess Nourah bint Abdulrahman University [PNU], Riyadh, Saudi Arabia; ^2^ Faculty of Medicine, University of Khartoum, Khartoum, Sudan; ^3^ Institut du Cerveau – Paris Brain Institute - ICM, Sorbonne Université, INSERM, CNRS, APHP, Paris, France; ^4^ CNRS, INCIA, Université de Bordeaux, Bordeaux, France; ^5^ Ecole Pratique des Hautes Etudes, EPHE, PSL Research University, Paris, France

**Keywords:** spastic paraplegia, clinical spectrum, genetic heterogeneity, phenotype-genotype correlation, molecular mechanisms, diagnostic yield, diagnostic gap, allelic variants

## Abstract

Hereditary spastic paraplegias (HSP) are a heterogeneous group of motor neurodegenerative disorders that have the core clinical presentation of pyramidal syndrome which starts typically in the lower limbs. They can present as pure or complex forms with all classical modes of monogenic inheritance reported. To date, there are more than 100 loci/88 spastic paraplegia genes (SPG) involved in the pathogenesis of HSP. New patterns of inheritance are being increasingly identified in this era of huge advances in genetic and functional studies. A wide range of clinical symptoms and signs are now reported to complicate HSP with increasing overall complexity of the clinical presentations considered as HSP. This is especially true with the emergence of multiple HSP phenotypes that are situated in the borderline zone with other neurogenetic disorders. The genetic diagnostic approaches and the utilized techniques leave a diagnostic gap of 25% in the best studies. In this review, we summarize the known types of HSP with special focus on those in which spasticity is the principal clinical phenotype (“SPGn” designation). We discuss their modes of inheritance, clinical phenotypes, underlying genetics, and molecular pathways, providing some observations about therapeutic opportunities gained from animal models and functional studies. This review may pave the way for more analytic approaches that take into consideration the overall picture of HSP. It will shed light on subtle associations that can explain the occurrence of the disease and allow a better understanding of its observed variations. This should help in the identification of future biomarkers, predictors of disease onset and progression, and treatments for both better functional outcomes and quality of life.

## Introduction

Hereditary spastic paraplegias (HSP) are a group of heterogeneous neurodegenerative disorders which are characterized by an insidiously progressive weakness and spasticity of the lower extremities as their core defining clinical features. They constitute the second most frequent motor neuron disease (MND) with an estimated prevalence of 3–10/100,000 in most populations (Noreau et al., 2014). HSPs are perfect models for understanding current challenges in inherited neurodegenerative disorders, as they overlap genetically with different neurogenetic diseases, although they were previously considered as completely separate entities (Harding, 1983).

### Classification of HSP

Classical classification of HSP was historically based on the clinical phenotype and the mode of inheritance (Harding, 1983). Recently, there has been an increasing tendency to involve the underlying molecular pathogenetic mechanisms in the categorization of HSP with multiple trials to establish classifications and groupings of identified HSP forms/genes based on the affected function (Lo Giudice et al., 2014; Noreau et al., 2014; Vallat et al., 2016; de Souza et al., 2017; Blackstone, 2018; Boutry et al., 2019a; Darios et al., 2020; Darios and Stevanin, 2020). More recently, machine-learning approaches were also used in their clinico-genetic stratification (Vavouraki et al., 2021). This group of diseases suffers from the fact that it gathers diseases in which spasticity/pyramidal signs are sometimes only part of the clinical phenotype such as some neurodevelopmental disorders due to *AP4n* genes mutations, and, at the other end of the spectrum, disorders in which the spasticity/pyramidal signs are the core and sometimes the only symptoms in patients, such as most SPG4 and SPG8 patients. In addition, spasticity and pyramidal signs are often observed in almost all neurogenetic conditions at some point of the disease evolution. A nosology restricted to HSP disorders will then be inefficient and a new nosology of all neurogenetic conditions is then necessary to take into account these now common observations in almost all neurogenetic conditions (Cassis et al., 2015; Synofzik and Schüle, 2017; Elsayed et al., 2019b).

All current classifications are therefore far from optimum, and are just used to give a clue about the disease and its clinical presentation and molecular basis.

### The Landscape of HSP Genetics

HSP shows remarkable genetic and clinical heterogeneity and is by far the most heterogeneous condition together with peripheral neuropathies. It is transmitted *via* all classical modes of monogenic inheritance which include autosomal dominant (AD), autosomal recessive (AR), recessive or dominant X-Linked (X-L), and mitochondrial/maternal transmission. The prevalence of AD HSP varies between 0.5 and 5.5/100,000 whereas that of the AR HSP ranges from 0.3 to 5.3/100,000 in hospital-based studies and 0.6 to 2.6/100,000 in multisource studies (Ruano et al., 2014). X-linked and mitochondrial inheritance are rare and mainly affect congenital cases; this is particularly the case with X-linked inheritance.

While AD HSP is more frequent in northern Europe, AR HSP predominates in highly consanguineous populations with one of the highest values reported in Tunisia, where it is probably attributed to the high rates of consanguinity in Northern Africa (Ben Hamida et al., 1986; Boukhris et al., 2008, 2009; Ruano et al., 2014).

To date, more than 100 loci/88 genes are known to be implicated in the pathogenesis of HSP. Of those, 83 clinical-genetic forms have the designation spastic paraplegia gene “n” or SPGn. However, there are more than 25 genes that have been reported to cause HSP but not assigned an SPG designation including the four mitochondrial genes (*MTND4*, *MTTI*, *MTCO3*, *MTATP6*) that were associated with HSP/HSP-like phenotypes. In 12 of the 83 SPG forms, the underlying gene/protein is not yet identified (Klebe et al., 2015; Tesson et al., 2015; de Souza et al., 2017; Boutry et al., 2019a).

Consanguinity has been of great help in identifying HSP causative genes since only 17 HSP subtypes show AD inheritance whereas the number of identified AR HSP subtypes is progressively increasing mounting to 53 HSP forms. X-L inheritance is relatively rare being reported in five forms only, all with recessive inheritance. SPG4 is the commonest AD HSP form in many populations, particularly in Caucasians. It accounts for 17–79% of AD HSP cases with monoallelic forms of *KIF1A* variant carriers, SPG3A and SPG31 coming next (Schüle et al., 2016; Burguez et al., 2017; Dong et al., 2018; Boutry et al., 2019a; Méreaux et al., 2021b). The collective frequency of SPG4 together with SPG3A amounted to 90% of the AD HSP in some series (Dong et al., 2018). On the other hand, SPG11 and SPG7 are the most frequent AR HSP subtypes followed by SPG15 and SPG5 (Ruano et al., 2014; Tesson et al., 2015; Boutry et al., 2019b; Coarelli et al., 2019; Du, 2021).

HSP can also be caused by mutations in “non-SPG” genes. The majority of the “non-SPG” forms have an original phenotype and HSP stands as an allelic presentation, frequently reported in a single family (Table 1). Although the non-SPG forms will be included in multiple sections of this article, the focus will be on the 83 SPG forms.

**TABLE 1:** Table summarizing HSP forms associated with non-SPG genes and the main allelic phenotypes associated with causative variants in these genes.

Gene	Disease		HSP Phenotype		HSP mode of inheritance		Families with HSP		Reference
*BICD2*	AD: Spinal muscular atrophy, lower extremity-predominant, 2A, and 2B [SMALED2]		Complicated HSP Amyotrophy		AD		One German Family, four patients		Oates et al. (2013)
*ACO2*	Optic Atrophy 9 [OA9] (AR) Infantile Cerebellar-Retinal Degeneration (AR)		Complicated HSP Intellectual disability and Microcephaly		AR		One Arab-Bedouin family two patients		Bouwkamp et al. (2018)
*RNF170*	Spastic ataxia		Complicated HSP		AR		Five families, 11 patients		Wagner et al. (2019)
			Spastic paraplegia andataxia						
*HACE1*	Neurodevelopmental disorders [Simplex, AR]		Complicated HSP		AR		Two Families [Pakistani: Five patients, German three patients]		Akawi et al. (2015); Hollstein et al. (2015)
			Spastic paraplegia and psychomotor retardation with or without seizures [SPPRS]				Two Families [two, three patients]		
*GAD1*	-		Complicated HSP		AR		Two Pakistani families [two, four patients]		Lynex et al. (2004)
			CPSQ1: Symmetrical spastic cerebral palsy						
*EXOSC3*	Pontocerebellar hypoplasia (AR)		Complicated HSP		AR		Two Families [Arab origin: four patients, Bangladesh: two patients]		Zanni et al. (2013); Halevy et al. (2014)
*ALS2*	Juvenile ALS (AR)		Complicated HSP		AR		Families from Algeria, France Saudi Arabia Pakistan Sudan, Italy		Eymard-Pierre et al. (2002); Racis et al. (2014); Wakil et al. (2014); Daud et al. (2016); Elsayed et al. (2016b); Helal et al. (2018); Simone et al. (2018)
			Infantile, ascending HSP (IAHSP)				Iran		
*LYST*	Chediak-Higashi (AR)		Complicated HSP		AR		One Japanese family		Shimazaki et al. (2014)
			Cerebellar ataxia and peripheral neuropathy						
			MRI: Cerebellar and Thoracic spinal cord atrophy						
			Laboratory Granulocytes: Peroxidase stained giant granules						
*TPP1*	Spinocerebellar ataxia Autosomal Recessive 7 (SCAR7)		Complicated HSP. Bulbar palsy, reduced intellectual function, seizures, neck dystonia		AR		One family		Kara et al. (2016)
	Childhood-onset Ceroid lipofuscinosis, neuronal, 2 (AR)		Brain MRI: Cerebral atrophy, thin corpus callosum. Temporary response to L-DOPA				Sporadic case		
*IFIH1*	Aicardi-Goutières syndrome 7 (AR)		Pure HSP		AR				Crow et al. (2014)
	Singleton-Merten syndrome 1								
*FAM134B*	Neuropathy, hereditary sensory and autonomic, type IIB (AR)		Complicated HSP		AR		One Turkish family and two Saudi families		Ilgaz Aydinlar et al. (2014); Wakil et al. (2018)
*CCT5*	Neuropathy, hereditary sensory, with spastic paraplegia (AR)		Complicated HSP		AR		One Moroccan family		Bouhouche et al. (2006)
*POLR3A*	Tremor–ataxia with central hypomyelination (TACH) leukodystrophy Hypomyelinating leukodystrophy (HLD7) with Ataxia and Hypodontia		Complicated HSP Ataxia Dental abnormalities		AR		Norwegian cohort with sporadic HSP		Rydning et al. (2019)
	Leukodystrophy, hypomyelination, 7, with or without oligodontia and/or hypogonadotropic hypogonadism/Wiedemann-Rautenstrauch syndrome (AR)		Head and neck titubation/dystonia						
*GRID2*	Spinocerebellar ataxia, autosomal recessive 18		Complicated HSP		AR Deletion		Sporadic case		Maier et al. (2014)
			Ataxia: Dysarthria, dysmetria						
			Frontotemporal dementia						
			Hand muscle wasting						
			Brain MRI: Cerebellar and Mesencephalic atrophy						
*DNM2*	Charcot-Marie-Tooth neuropathy Centronuclear myopathy		Complicated HSP		AD		One large Siberian kindred		Sambuughin et al. (2015)
			Distal muscular wasting						
			NCS: mild distal motor/sensory axonopathy						
*CCDC88C*	Autosomal recessive congenital hydrocephalus late-onset Spinocerebellar ataxia type 40 (AD)		Pure HSP		AD		One Sudanese family		Yahia et al. (2021)
*MTCO3*	Myopathy, Exercise intolerance, Encephalopathy, Lactic acidemia (one of the specific sporadic mitochondrial myopathy syndromes)		Complicated HSP		Mitochondrial		One family		Tiranti et al. (2000)
	Recurrent Encephalopathy Leber Hereditary Optic Atrophy (neuropathy) (LHON)		Developmental delay and Ophthalmoplegia				Sporadic Case		
*MTTI*	Cardiomyopathy Familial progressive necrotizing encephalopathy		Complicated HSP		Mitochondrial		One family		Corona et al. (2002)
	Leigh syndrome Myoclonus epilepsy, Progressive (MERRF) Mitochondrial Hypomagnesemia, hypokalemia, hypertension, and hypercholesterolemia		Dysarthria, severe hearing loss, mental regression, ptosis, ophthalmoparesis, and diabetes mellitus, cardiomyopathy						
*MTND4*	Leber optic atrophy (neuropathy) +/− dystonia		Complicated HSP		Mitochondrial		One family		Clarençon et al. (2006)
	MELAS Syndrome Late-onset encephalopathy		Visual loss, Sexual and Urinary disturbances, and visual evoked potentials: Abnormal						
*MTATP6*	Myopathy, Lactic Acidosis and Sideroblastic anemia 3 (MLASA3)		Complicated HSP		Mitochondrial		One family		Verny et al. (2011)
	Leigh syndrome		Developmental delay, retinal degeneration						
	Later onset: Bilateral striatal necrosis		NCV: Axonal neuropathy						

Over the last decade, and with the advent of next-generation sequencing (NGS)-based techniques, the genetic basis of HSP appears more complicated than previously thought. HSP forms with mixed inheritance (AD/AR) modes have been observed in six HSP forms. Moreover, a subtle mixed inheritance pattern with one dominating mode of inheritance and probable allele-dose-dependent variability in the expressed clinical phenotype was also demonstrated in some forms, adding to the genetic heterogeneity, and contributing to the complexity, of the whole picture. An example of the allele-dose-dependent variability was clear in the Kinesin encoding gene *KIF1C*, known to cause HSP type 58 (SPG58). *KIF1C* was shown to present with mild or subclinical dominant phenotype in heterozygous carriers, with the same mutations that resulted in more severe recessive phenotypes in their homozygous states (Oteyza et al., 2014). Other examples among the frequent HSP forms are related to recessive and dominantly inherited variants in *SPG7* and *KIF1A* (Klebe et al., 2012a; 2012b) with limited phenotype-genotype correlations (Coarelli et al., 2019; Pennings et al., 2020).

### Genetic Diagnosis: Approaches, Yield and Gap in Genetic Etiology

Despite the advances in genetic studies in the last two decades that resulted in a remarkable improvement of the genetic diagnostic yield, it seems that we are just seeing the tip of the iceberg. The overall diagnostic gap in HSP is currently estimated to be 40–70% on average with a smaller gap of 25–40% reported in some studies with specific ethnicities (Novarino et al., 2014; Morais et al., 2017; Bis-Brewer and Züchner, 2018; Koh et al., 2018) despite the use of combined approaches. The diagnostic yield seems to vary based on many factors that are not all well characterized. However, the approach used for genetic testing, the bioinformatic pipelines used in data analysis, the availability of DNA of other members of the family to confirm genotype-phenotype co-segregation, all seem to play roles. The types of the families included in the cohort [AD, AR or simplex/sporadic] also affect the genetic diagnostic capacity with lower yields obtained in cohorts of simplex cases. However, contradicting values have been observed (Schüle et al., 2016; Bis-Brewer and Züchner, 2018). The underlying genetic cause itself, and particularly when it is not easily analysed (novel unknown genes, variants in non-coding regions, oligogenicity), likely plays an additional role in lowering the genetic yield, together with the approach used, and its suitability to detect these types of genetic causes (Bis-Brewer and Züchner, 2018; Koh et al., 2018). Not only do these factors work individually, but also sometimes their combined interaction may result in failure to reach the genetic diagnosis in a family, and thus contributing to the genetic diagnostic gap in a cohort. The gap in the diagnostic yield of genetic studies using whole exome sequencing (WES), with the majority of non-coding region uncovered, is being gradually highlighted as exampled by the deep intronic *POLR3B* variant which was identified as a cause of spastic ataxia, then later being shown to account for around 3.1% of genetically unclassified AR and sporadic cases (Bis-Brewer and Züchner, 2018). This might also suggest that the heterogeneity of the phenotype can be due to, or, modified by, an unexplored non-coding region. This may question many of the results that we have about the underlying pathophysiologic mechanisms to date. NGS-based gene panels tailored to spastic disorders, movement disorders or Mendelian disorders (clinical exomes), are currently preferred for candidate gene screening approach (Méreaux et al., 2021b). However, occasionally Multiplex Ligation-dependent Probe Amplification (MLPA) and Sanger sequencing for selected genes, are performed to exclude the commonest genes causing HSP before using the NGS-based techniques. The broader the spectrum of genes covered, the higher the opportunity to identify novel HSP genes presenting with novel allelic phenotypes (Table 1). This is especially important with the remarkable overlap demonstrated in neurogenetic disorders lately (Synofzik et al., 2014; Chrestian et al., 2016; Kara et al., 2016; van de Warrenburg et al., 2016; Morais et al., 2017; Synofzik and Schüle, 2017; Elsayed et al., 2019b; Elert-Dobkowska et al., 2019). The genetic diagnostic yield using targeted gene panels and clinical exomes ranges from ≈15 to 30% when known common HSP genes have been excluded at a provisional screening. However, the yield steps up and may reach 50% or more when the panels/clinical exomes are used as the primary screening tool with common HSP genes included in them (Burguez et al., 2017; Morais et al., 2017; Elert-Dobkowska et al., 2019; Méreaux et al., 2021b). WES, and more recently genome sequencing, on the other hand, provides a better diagnostic yield of 30–60% (Novarino et al., 2014; Kara et al., 2016; Schüle et al., 2016; Bis-Brewer and Züchner, 2018). The diagnostic gap further decreases when using combined approaches tailored to the nature of the cohort (Kara et al., 2016; Koh et al., 2018). The search for rearrangements, particularly in *SPAST* and *SPG11*, using analysis of coverage in NGS-based techniques must be performed as such mutational mechanisms are relatively frequent (Méreaux et al., 2021b). Systematic reanalysis of negative panels or exomes should also be performed regularly as alignment and base-calling are constantly improving.

Very few reports have found phenotype-genotype correlations which elicit the impact of patient’s gender, the affected gene, and the nature of the mutation, in addition to other factors including ethnicity and genetic background. The impact of the patients’ gender in the phenotypic presentation has been pinpointed in a meta-analysis of 147 studies on HSP including nine of the most frequent AD HSP and AR HSP subtypes. This identified male predominance in certain subtypes [SPG7 (AR HSP) and SPG31 (AD HSP)] (Erfanian Omidvar et al., 2019). This was observed previously in SPG4 as well (Orlacchio et al., 2005b). Interestingly, a recent study identified significant lower penetrance in SPG4 female patients (Parodi et al., 2018b). Moreover, age at onset was shown to be modified by factors including ethnicity, the disease-causing genes, and the nature of the causative mutation itself (Parodi et al., 2018b; Newton et al., 2018; Erfanian Omidvar et al., 2019; Rodrigues et al., 2019). Missense mutations were found to be associated with earlier onset in SPG4 patients while the p.Ala510Val variant in *SPG7* was found to be associated with a later disease onset and a more ataxic clinical presentation (Parodi et al., 2018b; Coarelli et al., 2019). Similarly, missense mutations in *SPG11* are often associated with a later onset (Rubegni et al., 2015). Genetic modifiers have been reported to explain part of the variability in clinical presentation. Examples include the p.Ser44Leu and p.Pro45Gln variants in *SPAST* mutation carriers (Svenson et al., 2004) which were shown to have a real functional impact on spastin protein stability (Schickel et al., 2007; Pantakani et al., 2008). The p.Ser44Leu variant in *SPAST* was first reported as a cause of mild disease presentation then turned out to be a rare polymorphism acting as a phenotypic modifier (Lindsey et al., 2000; Svenson et al., 2004; Erichsen et al., 2007). Another example is the effect of deletions of *SPAST* that can sometimes extend to the nearby gene, *DPY30*, affecting the age at onset of SPG4 patients. This has particular importance as these deletions are relatively frequent in SPG4 (Beetz et al., 2006; Depienne et al., 2007; Newton et al., 2018). Finally, somatic mosaicism was shown to be a potential source of variant expressivity in *SPAST* mutation carriers (Angelini et al., 2021). However, the reduced penetrance of several variants in HSP genes still remains to be explained with the nature of the variants, their impact on protein expression/stability, the presence of compensatory protein or partners yet to be explored. This is apparently not yet the whole explanation of the great inter- and intra-familial variability of HSP patients especially with the digenic/oligogenic inheritance which is now emerging as a cause of a number of neurogenetic disorders including amyotrophic lateral sclerosis (ALS) and epilepsy. However, these models may be altered by the type of mutations as well as by the frequency of the suggested modifiers in the populations, and this might explain the effect of ethnicity and might also indicate an indirect effect of the environment over genetics (Kousi and Katsanis, 2015; Bis-Brewer and Züchner, 2018; Amin et al., 2021). Numerous studies have also suggested an impact of epigenetics in Parkinson’s, Alzheimer’s, Huntington’s diseases, cerebellar ataxias (CA), and ALS (Chestnut et al., 2011; Desplats et al., 2011; Chouliaras et al., 2013; Goodnight et al., 2019; Haertle et al., 2019; Jia et al., 2019; Lardenoije et al., 2019). A role for epigenetics in apoptosis was suggested in motor neuron pathology in ALS, with the upregulation of DNA methylase increasing DNA methylation which results in apoptosis (Chestnut et al., 2011). The role of epigenetics has also been studied in synaptic plasticity, in which decreased levels of DNA methylases, with associated synaptic degeneration, was observed in aging and degenerative disorders (Desplats et al., 2011; Chouliaras et al., 2013; Xylaki et al., 2019). However, the role of epigenetics in the HSP field, and its impact on the “expression” of the mutation, is still unclear. Identification of the factors affecting clinical presentation require international collaborative efforts in order to make this quest feasible in such rare genetic entities (Bis-Brewer and Züchner, 2018).

Ethnic differences manifest, at many levels, in the frequency of genes causing HSP, the predominant mode/s of inheritance, and the phenotypic presentation of genes. These differences reflect underlying variations which include the level of consanguinity, the ethnic genetic background, and the interaction with the environment, from which the effect of epigenetic modification of gene expression cannot be excluded. Many other factors involved are not yet well characterized. Multiple examples of these ethnic differences have been reported. For example, in many Western cohorts, *KIF5A* mutations (SPG10) account for about 10% of the complicated forms of AD HSP. Interestingly the first Asian (Japanese) case with a *KIF5A* mutation was identified only recently and broadened the clinical and electrophysiological phenotypic spectrum of *KIF5A* (Kaji et al., 2016). Remarkable examples also include the AR HSP forms that were first identified in highly consanguineous populations such as the *KIF1A* variants which were first identified in African and Palestinian families, and subsequently reported in a multitude of families from all over the world (Vecchia et al., 2021). On many occasions, genes which were the solely identified in some ethnic groups turned out later to be quite frequent causes of AR HSP; for example SPG11 and SPG15 (Boukhris et al., 2009). The impact of these phenomena needs to be carefully considered in many regions of the world, where there are not enough facilities for advanced genetic research. Interesting phenotypes and underlying genetics can be identified in these populations if studies of HSP and other neurogenetic disorders are performed using scientific methods properly adapted to the population under investigation. This has been illustrated in the infrequent neurogenetic studies that included families or cohorts from the exceedingly consanguineous African, Middle Eastern and Asian countries. Examples include Libya, Iraq, Syria, Egypt, Saudi Arabia, Sudan, Oman, Iran among others (Sridharan et al., 1985; Rainier et al., 2003; Abou Jamra et al., 2011; Alazami et al., 2011; Novarino et al., 2014; Elsayed et al., 2016a, 2016b, 2018; Ahmed et al., 2017; Yahia et al., 2018, 2021; Farazi Fard et al., 2019). The studies that included cohorts from these unexplored areas are increasing in number and despite their relative sparsity, they have contributed remarkably to the existing knowledge about HSP and other neurogenetic disorders. A good illustration is the work by Novarino et al. which reported 18 novel genes in a large set of consanguineous families from Middle East and North-Africa (Novarino et al., 2014). They illustrated the strong potential of digging into these virgin fields. Moreover, in many countries in the less developed regions of the world that are entirely unexplored from a genetic point of view, epidemiological data about most of genetic diseases are not available. This gives a hint about the fact that many of the current statistical estimates are inherently untrue. These population-based differences, highly stressed in recent research, show clearly that the overall portrait of the phenotypic presentation, the mechanisms and molecular pathways involved in the causation and pathogenicity of HSP, and the resulting cellular adaptations to the disease, will never be completely well-characterized if this gap is not overcome.

### Clinical Spectrum of HSP

#### Assessment and Diagnostic Approach of HSP Patients

The pivotal point that strongly suggests HSP is positive family history. However, HSP remains a diagnosis of exclusion in sporadic cases, where a number of other conditions such as HTLV1 infection, food intoxication, malformations, and cancer must be excluded through appropriate investigation. In particular, the assessment of the patient requires accurate history and clinical examination using the Spastic Paraplegia Rating Scale to assess the severity of the motor symptoms (Schüle et al., 2006). However, a variety of disability scores are also used for assessment of the patient’s disability stage. Further assessment including radiological, electrophysiological and laboratory testing, will give additional evidence on the nature of the disease and help to eliminate important differential diagnoses. The principal radiological assessments utilized are magnetic resonance imaging (MRI) of the brain and spinal cord. While MRI of the spine does not show significant abnormalities most of the time, brain MRI on the contrary (or sometimes computerized tomography (CT) scan of the brain) can give critical information that can assist in the exclusion of various differential diagnoses such as leukodystrophies and leukoencephalopathies, neurodegeneration with brain iron accumulation, ALS, or infectious and dysimmune causes. In addition, it will provide direction to the category of HSP (eg. HSP with thin corpus callosum (TCC)) and probable genes beyond it with the prominent example of *SPG11* being the most likely gene in TCC HSP. However, the question of the exclusion of leukodystrophies is now debated in light of the overlap of several HSP forms with this group of diseases.

Electrophysiological tests include electromyogram/nerve conduction studies (EMG/NCS), evoked potentials (somatosensory [SEP], visual, and auditory). These studies provide ample information about the underlying pathology and the clinical presentation of the patient resulting in both better diagnostic yield and delineation of the clinical spectrum of the disease phenotype.

### Associated Signs and Clinical Phenotypes

There is vast heterogeneity of the phenotypes associated with HSP. In this review, we derived clinical associations that may aid in a better understanding of HSP as a model of inherited neurodegenerative disorder. The core clinical presentation of patients is well-known to be with pyramidal syndrome which consists of increased muscle tone (spasticity with scissoring and clonus in its most severe form), hyperreflexia and extensor plantar response (Babinski sign). Classically, HSP can be pure (uncomplicated) or complex (complicated) according to the absence or presence of additional neurological and extra-neurological manifestations but this distinction has most of the time no relation to the genetic etiology. Indeed, with the recent surge of genetic data, and the expansion of the associated clinical picture of several mutated genes, some forms were found to manifest with a wider clinical presentation ranging from pure to complex, with differences sometimes within the same family. Moreover, some HSP genes were linked to multiple allelic presentations that included even non-HSP phenotypes (Elsayed et al., 2019a; 2019b). Pure forms can also present with clinical features suggesting involvement of the dorsal column, with diminished or even abolished vibration sense. Sphincter involvement (principally urinary urgency) can occur in pure HSP too due to increased bladder muscle tone (Fink, 1993, 2014; Finsterer et al., 2012; Lo Giudice et al., 2014; Klebe et al., 2015; Tesson et al., 2015; de Souza et al., 2017; Boutry et al., 2019b; Elsayed et al., 2019b). Complicating neurological features include most frequently deterioration of the cognitive/mental functions (>46 SPG forms). The cognitive function shows variable levels of alteration with the AR complicated HSP forms having a higher degree of impairment. A recent large meta-analysis study found no report of cognitive disability in SPG3A patients although it was often reported in SPG4, the two AD HSP forms that were initially thought to be mostly pure. However, the same meta-analysis study found that the frequency of intellectual disability in AR HSP patients to be highest in SPG54 (89%) followed by SPG11 (86%), SPG15 (78%), SPG35 (71%), and SPG7 (8%) respectively (Erfanian Omidvar et al., 2019). The cerebral palsy-like HSP forms associated with mutations in genes encoding members of the AP4 complex (SPG47, SPG50, SPG51, SPG52) have been associated with severe cognitive impairment in agreement with their role in vesicular trafficking during the development of the central nervous system. All mutations reported in literature in the four genes - *AP4B1*, *AP4M1*, *AP4E1*, *AP4S1* - were loss of function variants except for a missense in *AP4M1* gene, that was reported in a Greek family by Bettencourt et al., in which there was compound heterozygosity with a loss of function mutation in one patient (Verkerk et al., 2009; Abou Jamra et al., 2011; Moreno-De-Luca et al., 2011; Bauer et al., 2012; Tüysüz et al., 2014; Abdollahpour et al., 2015; Hardies et al., 2015; Bettencourt et al., 2017; Jacinto-Scudeiro et al., 2019). Cognitive impairment is almost a universal feature in all complicated X-L recessive HSP forms which may suggest that it should be considered in the differential diagnosis in the cases of developmental delay and cognitive impairment in males. However, the reliability of these observations is doubtful as X-L HSP is rare and only a few families have been reported. Adducted thumbs are probably of better help in gene prognosis, at least in *L1CAM* mutation carriers.

Peripheral neuropathy or amyotrophy (>40 HSP forms) is the second most frequent complicating feature, followed by cerebellar signs (>37 HSP subtypes), and eye signs (cataract, retinal/macular degeneration, strabismus, and optic atrophy) taken collectively (≈22 forms). Clinical presentation can be further complicated by bulbar or pseudobulbar palsy, psychiatric symptoms, auditory neuropathy, extrapyramidal signs, stereotypic laughter, or epilepsy (Table 2). Because of the frequent association of CA with the spasticity and the frequent occurrence of pyramidal signs in patients with CA as the main complicating clinical symptom, the concept of ataxia-spasticity spectrum emerged. Patients with pyramidal or cerebellar signs can present with a phenotypic presentation varying along a continuum of clinico-genetic entities from pure CA, to spastic ataxia and to pure HSP. Spastic ataxias refer to disorders in which most patients have a clinical presentation with equal contribution of pyramidal and cerebellar involvement such as in spastic ataxia of Charlevoix-Saguenay, and in SPG7, SAX2, SPG43 and *RNF170* mutation carriers ((Synofzik and Schüle, 2017). The list of clinical entities designated a spastic ataxia is progressively broadening with nine syndromes given the designation of spastic ataxia “n” [SPAX “n”] to date.

**TABLE 2:** Grouping of various HSP forms based on the clinical presentation.

Clinical category	HSP Forms
Pure/Complex HSP	AD: SPG4, SPG6, SPG8, SPG10, SPG12, SPG13, SPG19, SPG31, SPG33, SPG41, SPG42, SPG73, SPG80
	AR: SPG5A, SPG11, SPG15, SPG24, SPG27, SPG28, SPG45/65 (*NT5C2*), SPG56, SPG57, SPG58, SPG62, SPG76, SPG77, SPG80
	AR/AD: SPG3A, SPG7, SPG9, SPG18, SPG30, SPG72
	X-Linked recessive: SPG16, SPG34
Only Pure HSP reported	AD: SPG12, SPG13, SPG19, SPG41, SPG42
	AR: SPG24, SPG62, SPG83
	X-Linked recessive: SPG34
SP complicated with cognitive impairment/intellectual disability	AD: SPG4, SPG6, SPG10, SPG80
	AR: SPG5A, SPG11, SPG14, SPG15, SPG20, SPG21, SPG23, SPG26, SPG27, SPG32, SPG35, SPG39, SPG44, SPG45/65 (*NT5C2*), SPG46, SPG47, SPG48, SPG49, SPG50, SPG51, SPG52, SPG53, SPG54, SPG55, SPG56, SPG59, SPG60, SPG61, SPG64, SPG69, SPG75, SPG77, SPG78, SPG81, SPG82, *ABHD16A*
	X-Linked recessive: SPG1,SPG2, SPG16, SPG22
	AR/AD: SPG3A, SPG9, SPG18, SPG30
HSP complicated peripheral neuropathy [with/without amyotrophy]	AD: SPG4, SPG6, SPG10, SPG31, SPG36, SPG80
	AR: SPG11, SPG14, SPG15, SPG21, SPG23, SPG25, SPG26, SPG27, SPG28, SPG39, SPG43, SPG46, SPG48, SPG55, SPG56, SPG57, SPG60, SPG61, SPG66, SPG68, SPG74, SPG75, SPG76, SPG78, SPG79
	AR/AD: SPG3A, SPG7, SPG9, SPG30
HSP complicated with amyotrophy [no peripheral neuropathy]	AD: SPG8, SPG17, SPG38, SPG73
	AR: SPG5A, SPG18, SPG20, SPG35, SPG47, SPG51, SPG52, SPG63, SPG64, SPG65, SPG67, SPG70, SPG77
	AR/AD: SPG58, SPG72
HSP complicated with cerebellar signs (clinical) [with/without evidence of cerebellar atrophy on brain MRI]	AD: SPG4, SPG10, SPG31, SPG80
	AR: SPG5A, SPG11, SPG15, SPG20, SPG21, SPG26, SPG28, SPG35, SPG39, SPG44, SPG46, SPG48, SPG49, SPG59, SPG60, SPG62, SPG64, SPG67, SPG68, SPG75, SPG76, SPG77, SPG78, SPG79, SPG82
	AR/AD: SPG7, SPG9, SPG30, SPG58
	X-Linked recessive: SPG1, SPG2, SPG22
HSP complicated with extrapyramidal signs	AD: SPG10
	AR: SPG21. SPG35, SPG47, SPG48, SPG56, SPG78
	AR/AD: SPG58
	X-Linked recessive: SPG1, SPG22
HSP complicated with optic atrophy	AR: SPG35, SPG45/65 (NT5C2), SPG54, SPG55, SPG57, SPG68, SPG74, SPG75, SPG79, SPG82
	AR/AD: SPG7, SPG9
HSP complicated with cataract	AR: SPG26, SPG46, SPG64, SPG69
	AR/AD: SPG9
HSP complicated with strabismus	AR: SPG50, SPG54, SPG55, SPG77
HSP complicated with retinal/macular degeneration	AD: SPG10
	AR: SPG11, SPG15, SPG81
HSP complicated with deafness	AD: SPG10
	AR: SPG29, SPG46, SPG69
HSP complicated with anarthria	AD: SPG4
	AR: SPG35
HSP with bulbar features	AD: SPG10
HSP complicated with hypotonia	AR: SPG49, SPG51, SPG52
	X-Linked recessive: SPG22
HSP complicated with seizures/epilepsy	AD: SPG6
	AR: SPG35, SPG47, SPG50, SPG51, SPG77, SPG81, SPG82
	AR/AD: SPG18
	X-Linked recessive: SPG2
HSP complicated with stereotypic laughter	AR: SPG47, SPG50, SPG51, SPG52
HSP complicated with microcephaly	AR: SPG47, SPG49, SPG50, SPG51, SPG52, SPG64
HSP complicated with short stature	AR: SPG20, SPG27, SPG47, SPG52, SPG54, SPG56, SPG63
	AR/AD: SPG58
HSP complicated with developmental delay	AR: SPG20, SPG47, SPG51, SPG52, SPG53, SPG54, SPG56, SPG61, SPG69, SPG77, SPG81, SPG82, *ABHD16A*
	AR/AD: SPG58
HSP complicated with skeletal deformities	AR: SPG23, SPG27, SPG47, SPG49, SPG51, SPG52, SPG53, SPG59, SPG65, SPG66, SPG81
	AR/AD: SPG9
	X-Linked recessive: SPG1
HSP complicated with hypogonadism and infertility	AR: SPG46 [infertility in males]
	AR: SPG64 [delayed puberty]

HSP, hereditary spastic paraplegia. The modes of inheritance are indicated by: AD, AR, X-Linked recessive with AR/AD representing mixed inheritance.

**FIGURE 1:**
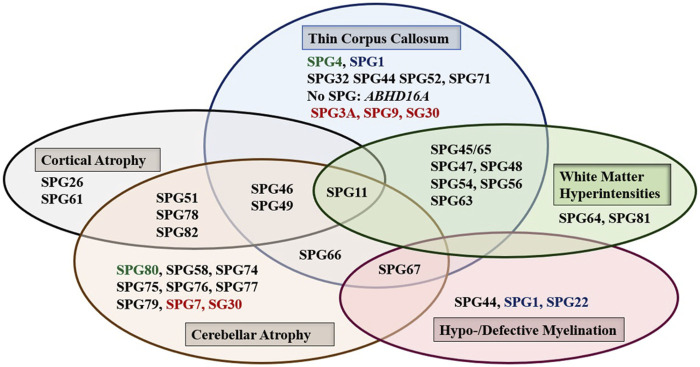
The main abnormal findings in MRI of the brain in different HSP subtypes. Font color codes correspond to various patterns of inheritance: AD HSP (green), AR HSP (violet), X-linked recessive (Blue), and Mixed AR/AD inheritance (red).

Brain, and to a lesser extent spinal cord, imaging abnormalities are frequent. MRI of the brain may show atrophy (generalized or focal: atrophy of the cerebral cortex and/or the cerebellum, and thin corpus callosum), features of dysmyelination (hypo- and de-myelination), leukodystrophic changes (white matter hyper signal intensity lesions), and rarely brain malformations. Although normal MRI is frequently encountered, abnormal radiological features are common and can be found in combinations or one at a time with great variability (Figure 1). It is interesting how the list of radiological features associated with HSP is constantly broadening as well. Recent reports detected MRI features mimicking the “eye of a tiger” sign, most commonly associated with pantothenate kinase e-associated neurodegeneration (PKAN), in SPG7 and the closely related spastic ataxia 5 (*AFG3L2*-mutated) patients (Calandra et al., 2020; Rizzo et al., 2020).

Extra-neurologic organs/systems involved include the eyes (cataract, strabismus), as well as the skeletal system, the heart, the skin, the hair and the gastrointestinal system.

The rapidly expanding genetic heterogeneity has led to an additional expansion of phenotypic heterogeneity, with the list of the reported complicating neurological and extra-neurological clinical features significantly expanding also. The spectrum of the HSP phenotype has broadened extensively to include further atypical disease presentations that were not reported previously in the earlier HSP cohorts (Harding, 1983) (Table 2). Non-exhaustive illustrative examples of atypical HSP presentations that make it worth reconsidering our working case-definition include tetraplegic cerebral palsy forms associated with AP4 complex mentioned earlier (Verkerk et al., 2009; Abou Jamra et al., 2011; Bauer et al., 2012), HSP associated with hyperbilirubinemia and persistent vomiting caused by hiatus hernia (SPG29) (Orlacchio et al., 2005a), atypical HSP presentation with areflexia, apnoea, hypoventilation, ventriculomegaly, and gastro-oesophageal reflux disease in association with SPG49/TECPR2 (Oz-Levi et al., 2012, 2013), and HSP with cutis laxa and cataract (SPG9) (Coutelier et al., 2015, 2016). On the other hand, some disorders which were considered as subtypes of HSP by the earlier HSP classification suggested by Harding in 1983, such as Sjögren-Larsson syndrome and Spastic Ataxia of Charlevoix Saguenay, are no longer classified as HSP in the recent classifications of neurogenetic disorders (Harding, 1983; Vallat et al., 2016; Bindu, 2020).

In addition to the above-mentioned examples, another dimension is highlighted by the phenotypes associated with the mitochondrial genes. MT-*ATP6* and *MT-TI* cause an HSP-like phenotype sometimes associated with cardiomyopathy (Verny et al., 2011), whilst *MT-CO3* has been linked to HSP but with Leigh syndrome-like lesions in the brain (Tiranti et al., 2000).

The greatest part of clinical heterogeneity can be attributed to the AR HSP forms which mainly present as complicated HSP in contrast to the tendency of AD HSP for pure, or relatively pure clinical presentations (Fink, 1993; Finsterer et al., 2012; Lo Giudice et al., 2014; Klebe et al., 2015; Lynch et al., 2015; Tesson et al., 2015; Elsayed et al., 2016b; de Souza et al., 2017; Boutry et al., 2019b); the more patients that are reported however, the more complex forms that are found. SPG4 and SPG11/SPG7 are the most frequent pure and complex HSP forms, respectively (Travaglini et al., 2018). These represent the commonest causes of AD HSP and AR HSP respectively in most reports (Ruano et al., 2014; Blackstone, 2018; Boutry et al., 2019a). AD HSP forms with complex presentation tend to be at low level of complexity with one or two complicating signs that are usually limited to peripheral neuropathy and amyotrophy except for SPG9, SPG29 and to a lesser extent SPG80 which show high level of complexity (Orlacchio et al., 2005a; Coutelier et al., 2015, 2016; Farazi Fard et al., 2019) (Figure 1; Table 2). On the other hand, the relatively rare X-L recessive HSP forms tend to present with complicated phenotypes except for SPG34, which was reported only in the pure form, and SPG16, which was found in pure and complex HSP forms.

### Age at Onset and Disease Progression

**FIGURE 2:**
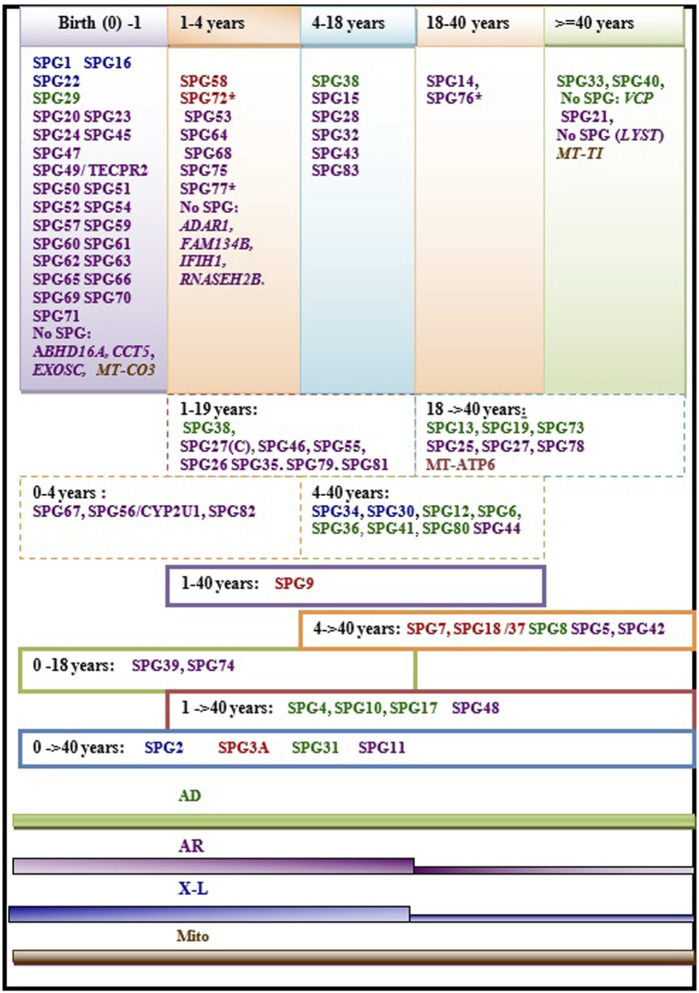
Regrouping of the age at onset of various HSP subtypes. The asterisk (*) indicates few exceptions in the age at onset range: SPG76*[one case is congenital] SPG72* and SPG77* has an age at onset range between (1–5 years)], SPG27 stands for the pure form and SPG27C for the complex form of SPG27. Font color codes represent the modes of inheritance: AD HSP (green), AR HSP (violet), X-linked recessive (Blue), Mitochondrial (brown), and Mixed AR/AD inheritance (red).

Age at onset of HSP is widely variable ranging from birth in some HSP subtypes to more than 40 years. In some rare cases the disease can have rather a late onset up to 76 years (Parodi et al., 1993; 2018a). The variability of age at onset does not only occur between the different forms but can also be observed within single HSP forms and even within families in patients carrying the same mutations (Figure 2). This is particularly the case in dominant forms (Figure 2) where heterogeneity is complicated by the incomplete penetrance. A meta-analysis study including a large set of HSP studies illustrated a significant association of ethnicity with differences in the age at onset in some HSP forms (Erfanian Omidvar et al., 2019), suggesting the influence of the genetic backgound. This study also identified earlier mean age at onset in *ATL1* mutated patients relative to patients with other AD HSP subtypes [SPG4 and SPG31] caused by mutations in *SPAST* and *REEP1*. Among the AR HSP subtypes included in the analysis, SPG35 showed a tendency for younger age at onset (Erfanian Omidvar et al., 2019).

It is often difficult to assess the age at onset in a precise way in HSP. This can be sometimes attributed to the subtlety of the presenting motor symptoms, especially in complex forms. In the pure HSP forms, several patients do not complain of the disease but clinical signs may be detected at examination (Hedera, 1993; Finsterer et al., 2012; Lo Giudice et al., 2014; Klebe et al., 2015; Tesson et al., 2015; de Souza et al., 2017; Boutry et al., 2019b). This may result in both underestimation and overestimation of the incidence and the prevalence of HSP.

We found that the age at onset of AR HSP tends to cluster in childhood in 80% (≈45 forms) of the genetic forms; however, two of the most frequent forms have relatively late onset (SPG5 and SPG7). More than 34 AR HSP forms together with three X-L recessive subtypes occur in toddlers (<5 years). AD forms do not show analogous clustering but tend to have variable age at onset. Overall, several HSP subtypes show considerable variability. Of these subtypes, with widely variable age at onset, are five AD HSP subtypes [including three of the commonest AD HSP forms (SPG4, SPG3A, SPG10)], two AR HSP (SPG11 and SPG48) and an X-L recessive HSP (SPG2) (Figure 2). In addition to these variations observed in the age at onset among different forms with different modes of inheritance, two large studies found a significant difference in age at onset based on the nature of the mutation in SPG4 patients, with earlier onset in patients with missense compared to those with truncating variants (Parodi et al., 2018b; Rodrigues et al., 2019).

Most HSP subtypes are slowly progressive, particularly at the start of the disease before they reach a static plateau without improvement (Hedera, 1993; Klebe et al., 2015; de Souza et al., 2017; Boutry et al., 2019a). However, substantial variability exists regarding the disease severity and its progress. Several forms deteriorate rapidly, while many subtypes show very slowly progressive course of disease so that they can be fairly considered as non-progressive. Surprisingly, late onset forms are more often associated with a more rapidly progressive evolution. This observation was supported by analysis of large HSP patient series (Schüle et al., 2016).

### Pathophysiology of HSP

#### Basic Pathophysiologic Mechanisms: HSPs Are Mainly Due to Neuronal Degeneration

The core phenotypic features of HSP result from axonal degeneration of neurons of the pyramidal motor system which is responsible for the voluntary movements in humans. The neurons of the pyramidal tracts extend from the layer V of the cerebral motor cortex and, after synaptic connection in the spinal cord with the secondary motor neuron, finally innervate the skeletal muscles at the neuromuscular junctions. Neurons are the principal category of cells that degenerate in HSP, even though the contribution of oligodendrocytes and other glial cells to the pathology of HSP cannot be ignored since several HSP genes are expressed in non-neuronal cells (Blackstone, 2012).

Neurons are polarized cells (Kevenaar and Hoogenraad, 2015) and the pyramidal tract neurons can be injured in a length dependent manner through a dying-back mechanism of their axons. Since the longest axons are more susceptible, this leads to the primary clinical involvement of the lower limbs (LLs) (Deluca et al., 2004; Blackstone, 2012, 2018). In the complex phenotypes, further regions of the central and peripheral nervous systems (CNS and PNS) alongside other extra-neurological organs and systems can also be involved. In these other tissues, the degeneration of cell bodies occurs in interneurons with shorter axons as well and then the dying-back hypothesis is less evident.

### Pathways Involved in HSP Pathogenesis

**FIGURE 3:**
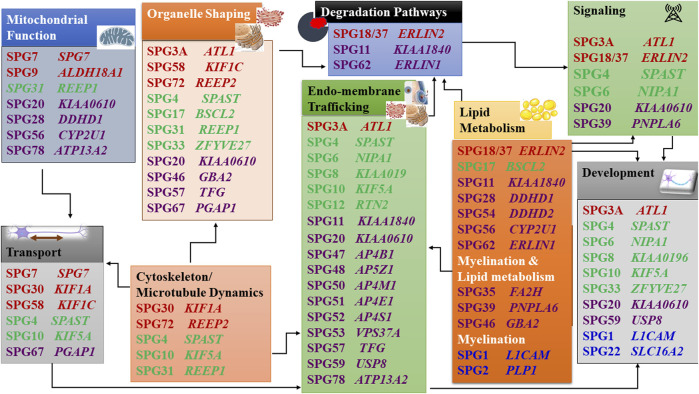
Intersections of HSP pathogenetic mechanisms highlighting the interplay between pathways. Font color codes correspond to various patterns of inheritance: AD HSP (green), AR HSP (violet), X-linked recessive (Blue), and Mixed AR/AD inheritance (red).

When the function of the affected HSP protein is known or could be inferred from their loss of function in models, they can be grouped into common cellular pathways based on ontology terms. In this article, we could distinguish 11 major functional themes (Supplementary Figure, Table 3). HSP genes encode proteins of known or suspected functions mainly in intracellular trafficking (membrane traffic and energy-dependent transport), organelle shaping, myelination, development, metabolism (predominantly lipid metabolism), recycling/degradation, cytoskeleton dynamics, mitochondrial functions, and signaling pathways. However, it recently emerged that dysfunction of one cellular organelle or function can have a deep impact on other cellular functions (Figure 3). For example, proteins implicated in the cytoskeleton dynamics such as spastin, can participate in organellar shaping particularly in ER morphogenesis, or modulate the formation of lipid droplets (LD) which can be considered as the intersection of these pathways with lipid metabolism in the pathogenesis of HSP (Figure 3) (Papadopoulos et al., 2015; Tadepalle and Rugarli, 2021). Furthermore, loss of spatacsin (SPG11) that promotes tubule formation on lysosomes also affects sphingolipid metabolism and leads to impairment of cholesterol recycling from lysosomes resulting in their accumulation and decrease in the cholesterol content of the plasma membrane which allowed us to include SPG11 as the 17th HSP form involving lipid metabolism (Boutry et al., 2018; 2019a).

**TABLE 3:** Table summarizing the 88 HSP clinical-genetic entities with special focus on the functions of their proteins and our suggested primary and secondary functional categories.

SPG code (Inheritance)	OMIM #/%	Gene locus	Age at onset	P/C	Protein	Function	Frequency	Reference
AD/AR HSP								
SPG3A (AD/AR)	#606439	*ATL1* 14q22.1	<1–51 (mainly <10) years	P/C	Atlasin GTPase1	Dynamin GTPase: ER shaping, ER and lipid droplet fusion, Inhibit BMP signaling	10% [39% of young-onset patients] AR: One family	Zhao et al. (2001)
SPG7 (AR/AD)	#607259	*SPG7*	4–42 years	P/C	Paraplegin	Component of the mitochondrial AAA protease	1.5–6%	Casari et al. (1998); Coarelli et al. (2019)
		16q24					7% of AR families Rare AD cases	
SPG9A (AD)	#601162	*ALDH18A1*	13–59 years	C	Pyrroline-5-carboxylate synthase (P5CS) protein	Enzyme: Pyrroline-5-carboxylate synthase with glutamate kinase (GK) and γ-glutamyl phosphate reductase activities (amino-acid metabolism)	Seven families	Seri et al. (1999); Coutelier et al. (2015)
		10q23.3-q24.1						
SPG9B (AR)	#601162	*ALDH18A1*	1–7 years	P/C	Pyrroline-5-carboxylate synthase (P5CS) protein	Enzyme: Pyrroline-5-carboxylate synthase with glutamate kinase (GK) and γ-glutamyl phosphate reductase activities (amino-acid metabolism)	Two families	Seri et al. (1999); Coutelier et al. (2015)
		10q23.3-q24.1						
SPG18 (AR)	#616586	*ERLIN2*	<2 years	AR: C	Erlin-2	ER-associated degradation pathway (ERAD)	Seven families [AR: Five families, AD: Two families]	Alazami et al. (2011); Yıldırım et al. (2011); Rydning et al. (2018)
SPG18 (AD)				AD: P				
(SPG37)		8p11.23						
SPG37 (AD)	% 611945	8p21.1-q13.3	8–60 years	P	Protein not identified	Protein not identified	One family	Hanein et al. (2007)
(AD SPG18)								
SPG30 (AR/AD)	#610357	*KIF1A*	10–39 years	AR: P/C	Kinesin-like protein KIF1A	Motor protein, axonal anterograde transport	17 families	Klebe (2006); Erlich et al. (2011); Esteves et al. (2014)
		2q37.3		AD: C				
SPG58 (AR)	#611302	*KIF1C*	2–4 years	P/C	Kinesin family member 1C	Motor protein; Axonal transport	Six families	Dor et al. (2014); Novarino et al. (2014)
	*603060	17p13.2				Retrograde Golgi to ER transport)		
SPG72 (AR/AD)	#615625	*REEP2*	3–4 years	P/C	Receptor expression-enhancing protein 2	ER membranous protein: ER shaping	AD: Two families	Esteves et al. (2014)
		5q31.2					AR: Two families	
AD HSP [gene identified]								
SPG4 (AD)	#182601	*SPAST*	1–80 years	P/C	Spastin	AAA protein: Microtubule dynamics (Microtubule severing), inhibits BMP signaling ER morphogenesis, Endosomal trafficking Cytokinesis LD biogenesis	28–50% [40% ] of AD	Hazan et al. (1999)
		2p22.3					9–18% of sporadic cases	
SPG6 (AD)	#600363	*NIPA1*	8–37 years	P/C	NIPA1/Non-imprinted in Prader Willi/Angelman syndrome 1	Mg2+ transporter: Inhibitor of BMP pathway, Endosomal trafficking	14 families	Chai et al. (2003); Rainier et al. (2003)
		15q11.2						
SPG8 (AD)	#603563	*KIAA0196*	10–60 years	P/C	Strumpellin	Cytoskeleton/Actin remodeling, Endosomal traffic	19 families	Valdmanis et al. (2007)
		8q24.13						
SPG10 (AD)	#604187	*KIF5A*	2–51 years	P/C	Kinesin heavy chain isoform 5 A	Motor protein: microtubule-dependent ATPASE, anterograde axonal transport	3%	Reid et al. (2002)
		12q13.3						
SPG12 (AD)	#604805	*RTN2*	7–24 years	P	Reticulon 2	ER shaping protein	Four families	Montenegro et al. (2012)
		19q13.32						
SPG13 (AD)	#605280	HSPD1	17–68 years	P	Heat shock protein 60 Kda protein 1/chaperonin	Mitochondrial chaperonin/Mitochondrial regulation	Two families	Hansen et al. (2002)
		2q33.1						
SPG17 (AD)	#270685	*BSCL2*	2–60 years	C	Seipin	ER scaffolding protein for lipid metabolism, lipid droplet biogenesis at ER	23 families	Windpassinger et al. (2004)
		11q12.3						
SPG31 (AD)	#610250	*REEP1*	Variable	P/C	Receptor expression-enhancing protein 1	ER-shaping protein, mitochondrial-ER interface functions, ER-microtubule interaction, LD regulation	4.5%	Züchner et al. (2006)
		2p11.2						
SPG33 (AD)	#610244	*ZFYVE27/Protrudin*	42**–**50 years	P	Protrudin	ER morphology protein, regulates and promotes protrusion and neurite outgrowth LE-ER contact, interactor of spastin	One family [it is debated because of the variant frequency]	Mannan et al. (2006)
DEBATED		10q24.2						
SPG42 (AD)	#612539	*SLC33A1*	4–42 years	P	Acetyl-coenzyme A transporter 1	Acetyl-CoA transporter (role in glycolipid metabolism) BMP signaling autophagy	One family	Lin et al. (2008)
		3q25.31						
SPG73 (AD)	#616282	CPT1C	19–48 years	P/C	Carnitine palmitoyl-transferase	Neuronal isoform of Carnitine Palmitoyltransferase-1c, Lipid metabolism	One family	Rinaldi et al. (2015)
		19q13.33				Lipid-mediated signal transduction; Number and size of lipid droplets		
SPG80 (AD)	**#**618418	*UBAP1* 9p13.3	juvenile	P/C	Ubiquitin-associated protein 1	Vesicular trafficking, regulator Proteasomal degradation of ubiquitinated cell-surface proteins	14 families	Farazi Fard et al. (2019)
						Sorting endocytic ubiquitinated cargo proteins into MVB		
AD HSP [gene not identified]								
SPG19 (AD)	607152%	9q33-q34	36–55 years	P	Protein not identified	Protein not identified	One family	Valente et al. (2002)
SPG29 (AD)	609727%	1p31.1-21.1	Infancy	C	Protein not identified	Protein not identified	One family	Orlacchio et al. (2005a)
SPG36 (AD)	613096%	12q23-24	14–33 years	C	Protein not identified	Protein not identified	One family	Verkerk et al. (2009)
SPG38 (AD)	612335%	4p16-p15	16–19 years	C	Protein not identified	Protein not identified	One family	Orlacchio et al. (2008)
SPG40 (AD)	No OMIM #/%	(locus within SPG3A)	adulthood	P/C	Protein not identified	Protein not identified		Subramony et al. (2009)
SPG41 (AD)	613364%	11p14.1-11p.2	Mean 17 ± 3 years	p	Protein not identified	Protein not identified	One family	Zhao et al. (2008)
AR HSP [gene identified]								
SPG5/SPG5A (AR)	#270800	*CYP7B1*	4–47 years	P/C	25-hydroxycholesterol 7-alpha-hydroxylase	Cholesterol metabolism Conversion of 27-OH-cholesterol to 3β, 7α-diOH-5-cholestinoic acid	7%	Tsaousidou et al. (2008)
		8q12.3						
SPG11 (AR)	#604360	*KIAA1840*	<1–33 years	P/C	Spatacsin	Lysosome biogenesis, autophagy endosomal traffic	[339 patients]	Hanein et al. (2007); Du (2021)
		15q21.1					15–21% [59% of AR]	
SPG15 (AR)	#270700	*ZFYVE26*	4–19 years	P/C	Zinc finger FYVE domain-containing protein 26 [Spastizin]	Lysosome recycling protein (biogenesis) cytokinesis, autophagy, endosomal traffic	4% [31 families]	Hughes et al. (2001); Hanein et al. (2008)
		14q24.1						
SPG20 (AR)	#275900	*KIAA0610*	Infancy	C	Spartin	Endosomal traffic, Cytokinesis, inhibit BMP signaling, Lipid droplet maintenance, and turnover, Mitochondrial Ca2+ homeostasis Association with microtubules and tubulin	Eight families	Patel et al. (2002)
		*Spartin*						
		13q13.3						
SPG21 (AR)	#248900	*ACP33*	Adulthood	C	Maspardin	Late Endosomal/trans-Golgi traffic	Two families	Simpson et al. (2003)
		15q22.31						
SPG23 (AR)	#270750	*DSTYK;* 1q32.1	Infancy	C	Dusty protein kinase, Dual serine/threonine, and tyrosine protein kinase	Induces both caspase-dependent and caspase-independent cell death	Three families	Blumen et al. (2003); Lee et al. (2017)
						Cell death regulation		
SPG26 (AR)	#609195	*B4GALNT1;* 12q13.3	2–19 years	C	Beta-1,4 N-acetylgalactosaminyl transferase 1	Enzyme: GM2 synthase, Ganglioside metabolism	Seven families	Wilkinson et al. (2005); Boukhris et al. (2013)
SPG28 (AR)	#609340	*DDHD1;* 14q22.1	7–15 years	P/C	Phospholipase A1, DDHD1	Lipid metabolism	Four families	Bouslam et al. (2005); Tesson et al. (2012)
						Keeps mitochondrial membrane phospholipid and function		
						Forms lipid messengers in ER		
SPG35 (AR)	#612319	*FA2H;* 16q23.1	2–17 years (One family late onset)	C	Fatty acid 2-hydroxylase	Fatty acid metabolism Myelin stability Hydroxylation of myelin galactocerebroside	28 families	Dick et al. (2008), Dick et al. (2010); Edvardson et al. (2008)
SPG39 (AR)	#612020	*PNPLA6;* 19p13.2	Infancy, adolescence	C	Neuropathy target esterase	Acetyl Co-A transported, BMP signaling	10 families	Rainier et al. (2008); Synofzik et al. (2014)
						Lipid metabolism, Involved in membrane curvature, Axonal maintenance, phospholipid homeostasis		
SPG43 (AR)	#615043	*C19orf12;* 19q12	7–12 years	C	Protein C19orf12	Mitochondrial protein with unknown functions	Three families	Meilleur et al. (2010); Landouré et al. (2013)
SPG44 (AR)	#613206	*GJC2;* 1q42.13	1st or 2nd decade	C	Gap junction gamma-2 protein	Oligodendrocyte connexin (intercellular gap junction channel	One family	Orthmann-Murphy et al. (2008)
						CNS myelination		
SPG45 (SPG65) (AR)	#613162	*NT5C2;* 10q24.32-q24.33	Infancy	P/C	Cytosolic purine 5′-nucleotidase	Hydrolyzes IMP in both purine/pyrimidine nucleotide metabolism	Two families	Dursun et al. (2009); Novarino et al. (2014)
SPG46 (AR)	#614409	*GBA2;* 9p13.3	1–16 years	C	Microsomal Non-lysosomal glucosylceramidase/Glucocerebrosidase 2	Ganglioside metabolism Conversion of glucosylceramide to free glucose and ceramide. Involved in sphingomyelin generation	12 families	Boukhris et al. (2010); Martin et al. (2013)
						Brain Myelination and CNS development		
SPG47 (AR)	#614066	*AP4B1;* 1p13.2	Birth	C	AP-4 complex subunit beta-1	Member of the trafficking endocytic adaptor protein complex 4	13 families	Abou Jamra et al. (2011); Bauer et al. (2012)
						Non-clathrin coating vesicular trafficking (brain development and function) Somatodendritic sorting and autophagy		
SPG48 (AR)	#613647	*AP5Z1;* 7p22.1	2–50 years (mainly adult)	C	AP-5 complex subunit zeta-1	Member of the trafficking endocytic Adaptor protein complex 5	18 families	Slabicki et al. (2010); Breza et al. (2021)
						Helicase function, involved in DNA repair response		
SPG49 (AR)	#615031	*TECPR2;* 14q32.31	Infancy	C	Tectonin beta-propeller repeat-containing protein 2	Positive regulator of autophagy	Three families	Oz-Levi et al. (2012)
SPG50 (AR)	#612936	*AP4M1;* 7q22.1	Infancy	C	AP-4 complex subunit mu-1	Member of the trafficking endocytic adaptor protein complex 4	Five families	Verkerk et al. (2009)
						Non-clathrin coating vesicular trafficking (brain development and function) Somatodendritic sorting and autophagy		
SPG51 (AR)	#613744	*AP4E1;* 15q21.2	Infancy	C	AP-4 complex subunit epsilon-1	Member of the trafficking endocytic adaptor protein complex 4	Four families	Abou Jamra et al. (2011); Moreno-De-Luca et al. (2011)
						Non-clathrin coating vesicular trafficking (brain development and function) Somatodendritic sorting and autophagy		
SPG52 (AR)	#614067	*AP4S1;* 14q12	Infancy	C	AP-4 complex subunit sigma-1	Member of the trafficking endocytic adaptor protein complex 4	Five families	Abou Jamra et al. (2011)
						Non-clathrin coating vesicular trafficking (brain development and function) Somatodendritic sorting and autophagy		
SPG53 (AR)	#614898	*VPS37A;* 8p22	1–2 years	C	Vacuolar protein sorting-associated protein 37A	Subunit of the ESCRT-I complex involved in intracellular (endosomal trafficking)	Two families	Zivony-Elboum et al. (2012)
						Maturation of MVB and the sorting of ubiquitinated membrane proteins into internal luminal vesicles		
						Retromer component		
SPG54 (AR)	#615033	*DDHD2;* 8p11.23	<2 years	C	Phospholipase DDHD2	Enzyme: Phospholipase (lipid metabolism)	Nine families	Al-Yahyaee et al. (2006); Schuurs-Hoeijmakers et al. (2012)
						May play role in synaptic organization and plasticity		
						Distribution of ER-Golgi proteins (vesicle trafficking)		
SPG55 (AR)	#615035	*C12orf65;* 12q24.31	2–7 years	C	Probable peptide chain release factor C12orf65, mitochondrial	Member of the mediated ribosome rescue system in mitochondria Mitochondrial protein synthesis, uncertain Function	Three families	Shimazaki et al. (2012); Tesson et al. (2012)
						Defective protein decreases oxidative phosphorylation complexes I, IV, and V		
SPG56 (AR)	#615030	*CYP2U1*; 4q25	<1–8 years	P/C	Cytochrome P450 2U1	Hydroxylation of long chain fatty acids [eg. arachidonic acid, docosahexaenoic acid (DHA)] and vitamin B2	1.5% [nine families]	Tesson et al. (2012)
SPG57 (AR)	#615658	*TFG;* 3q12.2	Infancy	P/C	Protein TFG	ER morphogenesis Vesicle biogenesis/transport between ER and Golgi	Six families	Beetz et al. (2013)
SPG59 (AR)	*603158	*USP8;* 15q21.2	Infancy	C	Ubiquitin carboxyl-terminal hydrolase 8	Deubiquitination enzyme	One family	Novarino et al. (2014)
						Trafficking and sorting of lysosomal enzymes		
						Endosomal morphology and organization		
SPG60 (AR)	*612167	*WDR48;* 3p22.2	Infancy	C	WD repeat-containing protein 48	Deubiquitination regulation	One family	Novarino et al. (2014)
						DNA damage repair		
						Lysosomal trafficking		
SPG61 (AR)	#615685	*ARL6IP1;* 16p12.3	Infancy	C	ADP-Ribosylation-Like Factor 6-Interacting Protein 1	ER morphology protein	Three families	Novarino et al. (2014)
						Protein transport		
						Anti-apoptotic		
SPG62 (AR)	#615681	*ERLIN1*	childhood	P	Erlin-1	ER-associated degradation	Three families	Novarino et al. (2014)
		10q24.31				Cholesterol homeostasis; Lipid raft-associated		
SPG63 (AR)	#615686	*AMPD2*; 1p13.3	Infancy	C	AMP deaminase 2	Enzyme: Deaminates AMP to IMP in purine nucleotide metabolism	Two families	Novarino et al. (2014)
SPG64 (AR)	#615683	*ENTPD1*	1–4 years	C	Ectonucleoside triphosphate diphosphohydrolase 1	ATPase hydrolyzes extracellular ATP and ADP to AMP	Two families	Novarino et al. (2014)
		10q24.1				Regulate purinergic transmission and modulate P2 receptor		
SPG45 (SPG65) (AR)	#613162	*NT5C2*	Infancy	P/C	Cytosolic purine 5′-nucleotidase	Hydrolyzes IMP in both purine/pyrimidine nucleotide metabolism	Two families	Dursun et al. (2009); Novarino et al. (2014)
		10q24.32-q24.33						
SPG66 (AR)	#610009	*ARSI*	Infancy	C	Arylsulfatase I	Hydrolyses sulfate esters	One family	Novarino et al. (2014)
		5q32				Hormone biosynthesis, Modulation of cell signaling; Degradation of macromolecules		
SPG67 (AR)	*611655	*PGAP1*	<1–4 years	C	GPI inositol-deacylase	Mature GPI biosynthesis	One family	Novarino et al. (2014)
		2q33.1				ER-to-Golgi transport of GPI-Anchor proteins (DAF)		
SPG68 (AR)	*604806	*FLRT1*	2–3 years	C	Fibronectin-like domain-containing leucine-rich transmembrane protein 1	Cell adhesion; Receptor signaling	One family	Novarino et al. (2014)
Allelic, or same, disorder: SPOAN		11q13.1				Fibroblast growth factor signaling		
SPOAN	#609541	*KLC2*	Infancy	C	Kinesin light chain 2	Motor protein/axonal transport	47 families	Macedo-Souza et al. (2005), Macedo-Souza et al. (2009); Melo et al. (2015)
Allelic, or same, disorder: SPG68	*611729	11q13. 2						
SPG69 (AR)	#609275	*RAB3GAP2*	<1 year	C	Rab3 GTPase-activating protein non-catalytic subunit	ER morphogenesis	One family	Novarino et al. (2014)
		1q41				Exocytosis of neurotransmitters and hormones; Neurodevelopment		
SPG70 (AR)	*156560	*MARS*; 12q13.3	<1 year	C	Methionine--tRNA synthetase, cytoplasmic	Cytosolic methionyl-tRNA synthesis	1 family	Novarino et al. (2014)
SPG71 (AR)	*615635	*ZFR*	Infancy	C	Zinc finger RNA-binding protein	Unknown	One family	Novarino et al. (2014)
		5p13.3						
SPG74 (AR)	#616451	*IBA57*	3–12 years	C	Putative transferase CAF17, mitochondrial	Mitochondrial iron-sulfur cluster (ISC) assembly machinery	One family	Lossos et al. (2015)
		1q42.13				Important for normal respiratory complexes I, II, and IV and the lipoate-containing mitochondrial enzymes		
SPG75 (AR)	#616680	*MAG*	Infancy	C	Myelin-associated glycoprotein	Cell adhesion molecule involved in myelin maintenance and glia-axon interaction	Two families	Novarino et al. (2014)
		19q13.12				Inhibits neurite outgrowth and axonal regeneration		
SPG76 (AR)	# 616907	*CAPN1*	13–39 years	P/C	Calpain 1 protease	Intracellular protease	50 families	Gan-Or et al. (2016); Méreaux et al. (2021a)
		11q13.1				Synaptic plasticity, restructuring; Axon maintenance and maturation		
SPG77 (AR)	#617046	*FARS2*	6 months–5 years	P/C	Mitochondrial phenylalanine--tRNA Synthetase 2	Class II aminoacyl-tRNA synthetases	Four families	Yang et al. (2016)
		6p25.1				Mitochondrial protein translation		
SPG78 (AR)	**#**617225	*ATP13A2*	Adulthood	C	Cation-transporting ATPase 13A2	ATPases transport inorganic cations and other substrates across cell membranes	Five families	Kara et al. (2016)
		1p36.13				Autophagy; Endolysosomal trafficking, Mitochondrial function		
SPG79 (AR)	#615491	*UCHL1*	Childhood	C	Ubiquitin carboxyl-terminal hydrolase isozyme L1	DNA damage response, Thiol protease of peptidase C12 family	Three families	Bilguvar et al. (2013)
		4p13				Has ligase and hydrolase activities that may play roles in proteasomal protein degradation, a process critical for neuronal health		
SPG81	#618768	*SELENOI*	Infancy	C	Ethanolaminephosphotransferase 1	Phosphatidylethanolamine synthesis	Two Families	Ahmed et al. (2017)
		*2p23*						
SPG82	#618770	*PCYT2*	<2 years	C	Phosphoethanolamine cytidylyltransferase	Phosphatidylethanolamine synthesis	Four families	Vaz et al. (2019)
		*17q25*						
SPG83	**#**619027	*HPDL*	0–15 years	P	4-Hydroxyphenylpyruvate Dioxygenase-like	Mitochondrial metalloenzyme	Four families	Husain et al. (2020)
		*1p34*						
AR HSP [gene not identified]								
SPG14 (AR)	#605229	3q27-q28	∼30 years	C	Protein not identified	Protein not identified	One family	Vazza et al. (2000)
SPG24 (AR)	%607584	13q14	Infancy	P	Protein not identified	Protein not identified	One family	Hodgkinson et al. (2002)
SPG25 (AR)	#608220	6q23-24.1	30–46 years	C	Protein not identified	Protein not identified	One family	Zortea et al. (2002)
SPG27 (AR)	609041%	10q22.1-q24.1	P: 25–45 years	P/C	Protein not identified	Protein not identified	Two families	Meijer et al. (2004)
			C: 2–7 years					
SPG32 (AR)	611252%	14q12-q21	6–7 years	C	Protein not identified	Protein not identified	One family	Stevanin et al. (2007a)
X-linked HSP [gene identified]								
SPG1 (recessive X-linked)	#303350	*L1CAM*	Congenital	C	Neural cell adhesion molecule L1	Cell adhesion and signaling protein involved in axonal guidance	Few families with HSP	Rosenthal et al. (1992); Jouet et al. (1994)
		Xq28				Myelination Neurite outgrowth Neuronal cell migration and survival		
SPG2 (recessive X-linked)	#312920	*PLP1*	Variable	P/C	Proteolipid protein 1	Major myelin component Oligodendrocyte progenitor cell migration	Few families with HSP	Saugier-Veber et al. (1994)
		Xq22.2						
SPG22 (X-linked with female having a mild thyroid phenotype only)	#300523	*SLC16A2*	Early infancy	C	Monocarboxylate transporter 8	Thyroid (T3) hormone transporter MCT8	Few families with HSP	Claes et al., (2000); Starling et al. (2002); Dumitrescu et al. (2004)
		Xq13.2						
X-Linked HSP (gene not identified)								
SPG16 (recessive X-linked)	300266%	Xq11.2	Early infancy	P/C	Protein not identified	Protein not identified	Two families	Steinmüller et al. (1997)
SPG34 (recessive X-linked)	300750%	Xq24-q25	16–25 years	P	Protein not identified	Protein not identified	One family	Macedo-Souza et al. (2008)

P, pure; C, complex; AD, autosomal dominant; AR, autosomal recessive.

Another example is SPG7, in which alteration of the axonal transport due to mitochondrial impairment resulted in axonal degeneration due to traffic jam as was observed in paraplegin-deficient mice (Ferreirinha et al., 2004). In addition, numerous proteins produced from SPG genes are currently found to be connected with multiple cellular functions and are known to interact with numerous proteins which are either involved in the same pathway (or inter-digitations of pathways) or share the same gene ontology function (Figure 3) (Supplementary Figure, Table 3). It is then likely that alteration of one given HSP protein will impact multiple cellular functions. An HSP network has been produced to connect all known HSP proteins to hundreds of other potential HSP proteins (Novarino et al., 2014). This demonstrates how far we stand back from understanding the mechanisms of these disorders and make phenotype-genotype correlations difficult to establish.

One of the first identified mechanisms to be related to HSP was the disruption of the axonal transport, which was supported by the identification of several HSP genes that encode kinesin chains (*KIF5A* and later, *KIF1A, KIF1C*), essential members of the anterograde microtubule-dependent axonal transport (Crosby and Proukakis, 2002; Reid et al., 2002). Trafficking in the cell is probably the most frequent alteration found in HSP and is dependant on microtubule dynamics and mitochondrial functions, two other often affected pathways in HSP. The correct trafficking of vesicles and organelles is essential for neurons as well as is the turn-over of membranes and signaling machinery degradation. There is an obvious overlap between these functions in some forms of HSP. Trafficking involving kinesins necessitates functional mitochondria which are affected functionally and/or structurally in SPG7, SPG31, and SPG56 (Goizet et al., 2011; Tesson et al., 2012; Shanmughapriya et al., 2015; Lavie et al., 2017; Wali et al., 2020).

Another aspect of the problem in HSP is the endomembrane and autophagy/lysosomal trafficking (Toupenet Marchesi et al., 2021) which are interconnected with the signaling and receptor recycling/degradation. Cells depend on a highly regulated and dynamic membrane trafficking system for proper intracellular distribution of proteins, lipids, and complex carbohydrates. This transport relies on membrane-bound vesicles and can occur within different organelles inside the cell, or through the plasma membrane to and from the extracellular environment. Numerous HSP proteins are implicated in membrane trafficking, including regulators of the vesicle and endosomal trafficking and proteins involved in the morphogenesis of the organelles involved, mainly the endoplasmic reticulum (ER). *ATL1* (SPG3A) encodes a dynamin-related GTPase, which is present in both the ER and the Golgi compartments. Disease-causing variants in this gene not only lead to altered vesicle trafficking between ER and Golgi but also to altered Golgi morphogenesis (Namekawa et al., 2007). *AP4B1* (SPG47), *AP4M1* (SPG50), *AP4E1* (SPG51), and *AP4S1* (SPG52) encode proteins that constitute the adaptor protein 4 (AP-4) complex, which mediates vesicle formation and sorting of cargo for vesicles included in the secretory and endocytic pathways (Abou Jamra et al., 2011; Hirst et al., 2013). *AP5Z1* (SPG48), which encodes one of the subunits of the adaptor protein 5 (AP-5) complex, was also found to be involved in endosomal sorting, and to interact with spatacsin (SPG11) and zinc finger FYVE domain-containing protein 26 (SPG15), involved in the recycling of lysosomes (Chang et al., 2014; Renvoisé et al., 2014; Hirst et al., 2015, 2018). Magnesium transporter NIPA1 (SPG6) is a neuron-specific transmembrane protein expressed in early endosomes, cell surfaces, and the Golgi. It co-localizes with atlastin-1, and both were characterized as binding partners, with knock-down of *NIPA1* expression resulting in a marked reduction of atlastin-1 in neuronal processes (Botzolakis et al., 2011). A rat model with a *NIPA1* causative variant showed accumulation of tubular-vesicular organelles with endosomal features starting at axonal and dendritic terminals, followed by multifocal vacuolar degeneration in the CNS and peripheral nerves (Watanabe et al., 2013). *WASHC5*, formerly known as *KIAA0196* (SPG8) encodes a subunit of the large protein complex WASH, which connects endosomes to the cytoskeleton, through interaction with vacuolar protein sorting-associated protein 35 (VPS35) (Harbour et al., 2012). Phospholipase DDHD1 (SPG28) and phospholipase DDHD2 (SPG54) are two enzymes making the link between membrane-sorting and lipid metabolism: DDHD2 localizes to the cis-Golgi and also to the ER-Golgi intermediate compartment suggesting that it facilitates membrane and vesicle fusion by the modification of membranes through phospholipid hydrolysis (Inoue et al., 2012; Schuurs-Hoeijmakers et al., 2012; Yadav and Rajasekharan, 2016). *TECPR2* (SPG49) brought a new pathway to the HSPs, linking autophagy with the disease. Autophagy is a complex process responsible for the transport and degradation of cytoplasmic components in the lysosomes, which include compromised proteins and organelles. *TECPR2* encodes a member of the tectonin β-propeller repeat-containing family, containing both TECPR and tryptophan-aspartic acid repeat (WD repeat) domains. *SPG11* and *ZFYVE26* (SPG15) encoded proteins are also linked to autophagy due to their role in autophagic lysosome reformation (Chang et al., 2014).

Microtubules dynamics is highly related to organellar shaping and transport. This can be well illustrated by the role that the microtubules perform in the neurons. Microtubules keep the organellar organization thus maintaining the shape of these subcellular organelles (Figure 3). They also perform a vital role in neuronal development through their function in polarized axonal transport. Spastin (SPG4) is a key element of the microtubule dynamics, important for axonal growth, cellular fluidity and organellar structure (Errico et al., 2002; Butler et al., 2010; Solowska et al., 2014; Penazzi et al., 2016; Jeong et al., 2019). Atlastin (SPG3), RTN2 and REEP1/2 (SPG31, SPG72) are good illustrations of the implication of an abnormal shaping of the ER in HSP (Park et al., 2010; Esteves et al., 2014). The ER is a multifunctional organelle involved in several essential tasks for the cell, being the most abundant membrane compartment. It is responsible for the synthesis, modification, quality control and trafficking of secreted and integral membrane proteins and has a role in the Ca^2+^ regulation, lipid and sterol biosynthesis and distribution, carbohydrate metabolism, peroxisome biogenesis, and the formation of enzymes involved in drug detoxification. Additionally, ER interacts with several other membranes, including mitochondria, the Golgi apparatus, the plasma membrane, and with LDs.

Causative variants in *ATL1* (SPG3A) are the second most frequent cause of AD HSP. This gene encodes the protein atlastin-1, a member of the superfamily of dynamin-related GTPases that localize predominantly to the tubular ER and mediate the fusion of ER tubules (Hu et al., 2009; Orso et al., 2009). *RTN2* (SPG12) encodes reticulon 2, which also localizes to the ER tubules and that acts together with DP1/Yop1 as curvature-stabilizing proteins (Voeltz et al., 2006). *REEP1* (SPG31) and *REEP2* (SPG72) are part of the REEP/DP1/Yop1p family of ER-shaping proteins, which like RTN2 protein has two hydrophobic domains that, when inserted into the phospholipid bilayer, are thought to form hairpin loops generating a curvature in the ER membrane. Spastin interacts with atlastin-1, REEP1 and reticulon two proteins and they all seem to contribute to the shaping of the tubular ER network (Voeltz et al., 2006; Shibata et al., 2010; Beetz et al., 2013; Esteves et al., 2014). The microtubule-based motility in the ER is important for the proper organization and distribution of the ER tubules and the impairment of this link between the tubular ER and the microtubule cytoskeleton seems to be one of the HSP pathogenic mechanisms (Blackstone, 2012). Pathogenic *SPAST* variants (SPG4) affecting the AAA domain lead to constitutive binding to microtubules and then a dominant negative effect, and a mutant version of the M1-spastin isoform, specific to motor neurons, seems toxic by altering dynamic trafficking (Errico et al., 2002). However, there has been a debate for years about the effects of variants in *SPAST*. *SPAST* haploinsufficiency is the most prevalent opinion because of the existence of the truncating variants and because mutant spastin has not been detected on patients’ cells when tested. However, SPAST KO in the mouse does not reproduce the disease. In addition, a mouse model overexpressing at least one missense mutation in spastin has a phenotype at the heterozygous state with accumulation in neurites and decreased microtubule stability (Solowska et al., 2010, 2014)). We can postulate that haploinsufficiency contributes to neuronal vulnerability while some missense mutations act also by additional toxicity. Other genes whose proteins are associated with ER morphogenesis include *TFG* (SPG57). The TFG protein is important for the organization of transitional ER and ER exit sites into larger structures and variants in this gene lead to a disruption of the peripheral ER tubules organization and collapse of the ER network (Beetz et al., 2013; Elsayed et al., 2016b; McCaughey et al., 2016). *BSCL2* (SPG17), *ERLIN1* (SPG62), and *ERLIN2* (SPG18) encode proteins that also localize to the ER and relate to both ER and lipid metabolism pathways due to the key function of the ER in the synthesis, metabolism, and distribution of lipids and sterols (Blackstone, 2012). *BSCL2* encodes an ER protein thought to act at the interface of the ER with LDs, regulating their size. *ERLIN2* (SPG18) and *ERLIN1* (SPG62) encode proteins that localize preferentially to cholesterol-rich domains including lipid rafts, and are involved in the regulation of cellular cholesterol homeostasis. Additionally, ERLIN proteins are associated with ER-associated degradation (ERAD) that regulates protein degradation by the ubiquitin-proteasome machinery (Browman et al., 2006; Huber et al., 2013).

Mitochondria are very important organelles, that in addition to their role in producing ATP, are involved both in the response mechanisms to oxidative stress and apoptosis and are a source of toxic-free radicals. In HSP, at least 10 genes were already associated with mitochondrial dysfunction. *SPG7* encodes a protein, that together with *AFG3L2*, the gene which has been associated with Spastic ataxia 5 (AR) and Spinocerebellar ataxia 28 (AD), form the m-AAA metalloprotease complex of the inner mitochondrial membrane. This protease functions in protein quality control and is necessary for the regulation of ribosomal assembly (Atorino et al., 2003; Nolden et al., 2005). Fibroblasts from SPG7 patients showed reduced activity of complex I in the mitochondrial respiratory chain, and increased sensitivity to oxidative stress with increased mitochondrial DNA damage (Wedding et al., 2014). In a mouse model for SPG7, mitochondrial morphological abnormalities were observed in the synaptic terminals and distal regions of axons, correlating with the onset of motor impairment, and prior to axonal swelling and degeneration. The axonal swelling occurred due to a huge accumulation of organelles and neurofilaments, suggesting impairment of anterograde axonal transport, which may indicate that the axonal transport impairment could be secondary to mitochondrial dysfunction (Figure 3) (Ferreirinha et al., 2004; Rugarli and Langer, 2006). *CYP2U1* (SPG56) encodes an enzyme involved in lipid metabolism, which seems to have a folate-dependent neurodevelopmental component followed by a deleterious impact on mitochondrial bioenergetic function with patients’ fibroblasts showing alteration of mitochondrial architecture and bioenergetics with increased oxidative stress (Tesson et al., 2012; Pujol et al., 2021).

Lipid metabolism was recently identified as a mechanism highly affected in HSP, quite predictable, as the brain is highly enriched in lipids. Lipid metabolism is affected directly in 17 different entities and their number is constantly growing. The involvement of lipids in HSP pathogenicity and neurodegeneration can be through disturbed lipid metabolism or lipid distribution affecting various subcellular organelles and their function. However, this is not the whole story; as accumulating evidence also shows that HSP genes affecting various subcellular compartments can also alter lipid metabolism. This might make the 17 SPG forms involving disturbed lipid metabolism in our classification (Supplementary Figure, Table 3) just the tip of an iceberg. Furthermore, there is a remarkable number of genes/proteins which directly involve lipid metabolism causing other neurogenetic disorders with HSP-like presentation or HSP (as an allelic presentation). This overlap zone includes a broad spectrum of spastic (non-HSP) neurogenetic entities and a remarkable variety of underlying genes: *CYP27A1* [cerebrotendinous xanthomatosis (CTX)], *SERAC1* (AR: 3-methylglutaconic aciduria with deafness, encephalopathy, and Leigh-like syndrome), *PLA2G6* (a spectrum of AR neurodegenerative disorders including: HSP, infantile neuroaxonal dystrophy (INAD), and neurodegeneration with brain iron accumulation, parkinsonism (NBIA), *GALC* [AR: galactosylceramide lipidosis (Krabbe disease), *ARSA* [AR: metachromatic leukodystrophy (MLD)], *ABCD1* **[**X-linked Adrenoleukodystrophy (X-ALD)**]**, *ELOVL1* [AR: ichthyotic keratoderma, spasticity, hypomyelination and dysmorphic features (IKSHD)], *ELOVL4* [AR: ichthyosis, intellectual disability and spastic quadriplegia (ISQMR); AD: SCA34, Stargardt disease 3 ((STGD3)], *ALDH3A2* [Sjögren-Larsson Syndrome (SLS)]. This long list of examples provides clear evidence that the existing phenotypic classes of neurogenetic disorders need to be reconsidered (Darios and Stevanin, 2020; Tadepalle and Rugarli, 2021). Various types of lipids are involved in spastic neurodegeneration including precursor lipids as cholesterol and fatty acids as well as complex lipids like phospholipids and sphingolipids. Disturbed biosynthesis and degradation of these lipids can lead to either deficiencies of molecules with functional importance, or pathogenic accumulation of precursors or undegraded damaged molecules with toxic effect, all resulting in neurodegeneration (Darios and Stevanin, 2020; Tadepalle and Rugarli, 2021).

Lipids play many different roles from storing energy, to electrical insulation in myelin, and modulation of cellular calcium signaling and other signal transduction cascades. Additionally, they are important structural components of cell membranes. The composition of the membranes in lipids and sterols controls membrane fluidity, as well as compartmentalization into specialized microdomains and functions. Altered enzymatic activities is the predominant mechanism mediating the involvement of disturbed lipid metabolism in HSP pathogenesis, facilitating the identification of biomarkers and therapeutic targets. The first gene to be associated with lipid metabolism was *CYP7B1* (SPG5), which codes for a 7α-hydroxylase involved in cholesterol metabolism. More precisely, it works in two pathways: the “alternate/acidic pathway,” and the conversion of the cholesterol derivative dehydroepiandrosterone (DHEA) into 7-OH-DHEA. Additionally, there is growing evidence to suggest that this DHEA-related neurosteroids act as neuroprotective factors, and can reduce ischemia-induced neurodegeneration, which could relate *CYP7B1* disease-causing variants with a loss of this neuroprotective role (Tsaousidou et al., 2008). In addition, the enzymatic blockage of the pathway by the mutated CYP7B1 enzyme led to the accumulation of accumulation of CYP7B1 substrates (three oxysterols) including 25- and 27-hydroxycholesterol [25- and 27-hydroxysterol (27-OH)] in cerebrospinal fluid and serum(Schüle et al., 2010), which were demonstrated to be toxic to neurons in cultures (Schöls et al., 2017).

The development and maintainance of axons and myelin are also affected. This is particularly well illustrated by a pathway linking multiple HSP genes: the BMP signaling cascade. BMP signaling is essential for normal axonal function and axonal guidance (pathfinding), growth, and differentiation in the nervous system in mammals and other smaller models (eg. Drosophila) implying an important impact on the pathogenesis of the axonal degeneration. Numerous HSP proteins influence the BMP signaling pathway; these include Spastin (causing SPG4, the commonest AD HSP) in addition to Magnesium transporter NIPA1 (SPG6, AD), Maspardin (SPG20, AR), Spastizin (SPG15, AR), Atlastin (SPG3A, AD/AR), and PNPLA6 (SPG39), influence the BMP signaling pathway (Charron and Tessier-Lavigne, 2007; Wang et al., 2007, 2014; Wen et al., 2007; Tsang et al., 2009; Fassier et al., 2010; Bayat et al., 2011; Hegarty et al., 2013; Nahm et al., 2013; Song et al., 2013). Some of these diseases are late-onset and this suggests that abnormal development may contribute to the sensitization of the neuronal connections which will exert their deleterious action later in life. On the other hand, neural development itself, including myelination, is affected in multiple HSP forms as shown by abnormal branching of axons in animal models (Martin et al., 2013). For some forms, the age at onset implies the direct involvement of improper development in the pathology such as in AP4 syndromes (Behne et al., 2020) or SPG11 (Stevanin et al., 2007b; Pozner et al., 2020). Another example is given by the recent study showing partial phenotypic recovery of the mouse model of SPG56 using folate supplementarion during fetal life (Pujol et al., 2021).

Are there any possible phenotype-genotype correlations that can be suggested?

Despite the marked inter-digitation of pathways and contribution of a number of HSP proteins in multiple pathways, our functional classification (Supplementary Figure) allows to observe a few connections between the pathogenic pathways and the clinical presentation, age at onset and patterns of inheritance, that can help in solving a small part of the HSP puzzle. Overall, AR forms are observed to involve principally the metabolic pathways (lipids and nucleotide metabolism), in addition to the degradation pathways, myelination and the endomembrane trafficking, in agreement with the need for a complete loss of function to alter the pathway. On the other hand, AD HSPs are largely clustering in the microtubule dynamics, organelle shaping, development, and active cellular transport (often with mixed transmission) functional categories. Age at onset is not a discriminant of the altered functions except for some tendency for childhood-onset in forms related to lipids (11/17) and nucleotides (3/3) metabolism. Early childhood-onset was also observed in 9/19 forms affecting endomembrane trafficking which is probably because of their suggested roles during development. It is worth noting that all nine forms associated with the degradation pathway, present with cerebellar ataxia. The link between cerebellar syndrome and the degradation pathways is well known in the pathogenesis of AD cerebellar ataxias. Degradation of cellular inclusions is the basis of numerous subtypes of hereditary ataxia (HA) especially the polyglutamine AD cerebellar ataxias (PolyQ: SCA). In these disorders, the proteasomal and lysosomal degradation pathways stand as the chief cellular defense mechanisms against the aggregates (the hallmark of the polyQ disorders) (Seidel et al., 2015; Mori et al., 2016). Cognitive decline and intellectual disability were reported in forms that clustered in the functional categories with an influence on endomembrane trafficking [17/19 forms], myelination [7/7 forms], lipid metabolism [11/17 forms], and degradation pathways [8/9]. It is worth noting that although SPG3A affects endomembrane trafficking with no previous association with cognitive impairment, recent studies started to detect cases with cognitive decline from different ethnic backgrounds (Fusco et al., 2012; Terada et al., 2013; Erfanian Omidvar et al., 2019). This again points to the constantly evolving capacities of the possible phenotype-genotype correlations in HSP.

### Therapeutic Options and Opportunities

For all patients except those who are dopa-responsive, rehabilitation therapies, such as physical or speech therapy are the main proposed therapy. Physical therapy is based on passive tendon stretching, gait, and equilibrium rehabilitation. According to the functional repercussion of spasticity, medications used to decrease spasticity such as oral baclofen, intramuscular botulinum toxin, or intrathecal baclofen are often used. Orthopedic solutions such as special shoes for pes cavus, or Achilles tenotomy for equinovarus are also proposed to allow a longer autonomous gait. Sphincter disturbances should be confirmed by urologists and possible treatment options include oxybutynin chlorhydrate or trospium chlorure. Social solutions are proposed when patients cannot manage themselves anymore. Dancing and exergames are also proposed to preserve as much as possible motor and gait capacities (Synofzik and Ilg, 2014).

There is no curative treatment available yet for HSP patients. When dealing with rare diseases, one might want to find a common drug target for multiple HSP forms. The question which immediately arises is if there is a unifying theme. All functional subcategories are overlapping and interplay together. Lipid metabolism is important for membrane trafficking and signaling. Trafficking is dependent on ATP production provided by mitochondria. Organelle shaping is performed along cytoskeleton, and organelle functions are dependent on their shaping and trafficking. Animal models, and thus probably patients, show functional consequences of multiple pathways such as the accumulation of membranes along axons in the *SPG7* KO mouse modeling the loss of function of a mitochondrial protein (Ferreirinha et al., 2004) or the abnormal mitochondrial network in SPG56 patients in which an enzyme of the lipid metabolism is affected (Tesson et al., 2012). The interplay between all these functional pathways makes it theoretically feasible to find drugs that could be active in multiple HSPs.

In practice however, multiple pathways are probably affected in all HSP, which makes it difficult to find a common cure to all aspects of the disease efficient in multiple HSP subtypes.

Another bottleneck in drug development is the lack of biomarkers available. They may play an important role in helping in the monitoring of disease progression and response to treatment in clinical trials and preclinical testing in animal models. There are very few diagnostic non-clinical and laboratory-based findings - hormonal disturbances, biochemical changes, and accumulation of various metabolites - which are associated with specific phenotypes in several instances (Table 3). We can cite the accumulation of 27- and 25-hydroxycholesterol in CSF and blood of SPG5 patients (Schöls et al., 2017; Marelli et al., 2018), testosterone levels in blood in SPG46 males (Martin et al., 2013), levels of plasmatic amino-acids in *ALDH18A1* (SPG9) variant carriers (Coutelier et al., 2015), abnormal etherlipid profile in SPG82 (Vaz et al., 2019), brain imaging volumetry in SPG11 (Cardozo-Hernández et al., 2020), Coenzyme Q10/neopterin and brain calcifications in SPG56 (Pujol et al., 2021), and abnormal lipid peak at cerebral resonance spectroscopy in *DDHD2* (SPG54) mutation carriers (Schuurs-Hoeijmakers et al., 2012). Most of them are found in clinico-genetic entities affecting directly the lipid metabolism (Darios and Stevanin, 2020) but the abnormal lipid profiles in SPG11 (Boutry et al., 2018; 2019b) show that the lipid homeostasis is critical for proper functioning of most cellular functions in neurons.

Numerous preclinical studies on animal models have given promising results. They include drugs which target microtubules and act to regulate their stability in SPG4 models (Trotta et al., 2004; Orso et al., 2005) and in iPSC models of SPG3 (Zhu et al., 2014). Replacement therapies gave interesting results to compensate for loss of function, including the intracerebral injection of *CYP7B1* mRNA in SPG5 mimicking mouse models (Hauser et al., 2019) or viral delivery of proteins such as paraplegin in SPG7 models (Pirozzi et al., 2006) and spastin in neurons derived from iPSC of SPG4 patients (Havlicek et al., 2014). Among forms affecting directly the autophagy and lysosomal pathways, miglustat was succefully used to reduce ganglioside accumulation in SPG11 zebrafish models (Boutry et al., 2018), and tideglusib, a GSK3b inhibitor, prevented the neurite pathology in brain organoids of SPG11 patients (Pozner et al., 2018). Autophagy regulators might also be of interest in SPG48 (Breza et al., 2021) and rapamycin partially reversed the phenotype induced by Atlastin-1 loss of function in flies (Xu et al., 2017). In zebrafish mimicking SPG3, the modulation of the BMP pathway was also of interest to rescue the motor phenotype (Fassier et al., 2010). Even more promising data include the FDA approved molecules/drugs acting to decrease ER stress in SPG4 and SPG31 (Vaccaro et al., 2013; Julien et al., 2016). Folate supplementation was succefully used to improve the cognitive deficit in a SPG56 mouse model (Pujol et al., 2021). Finally, cholesterol-lowering drugs such as statins have been used in clinical trials in SPG5 patients but failed to reduce significantly the toxic 27-OH cholesterol in the CSF vs plasma (Schöls et al., 2017).

## Conclusion: At the Crossroads of Molecular Pathways and Clinical Options

It is still crucial to improve the identification of the genetic causes of HSP for various reasons. First, it provides the patient with a conclusive explanation, giving closure to an often long diagnostic wandering. Furthermore, once the gene, mutation, and transmission mode have been identified, it allows for accurate genetic counselling. Another prominent reason is that discovering all genes involved is important for the identification of unifying mechanisms, which offer better hope of treatment options because they increase the number of patients available. In addition, other approaches may be useful for treatment options and may result from understanding the clinical heterogeneity. Very few initiatives have been reported to search for its aetiology. Understanding the links between the various clinical presentations and the affected molecular pathways may well be useful for better deciphering of the mechanisms implicated in the causation of HSP. This will help identify the suitable biomarkers to be used and shed light on the possible predictors of disease onset, progression and guide the choices of treatment options, for better functional outcome and quality of life.
