# Supplementary material for: Insights into Clinical, Genetic, and Pathological Aspects of Hereditary Spastic Paraplegias: A Comprehensive Overview
Source: Front Mol Biosci. 2021 Nov 26;8:690899. doi: 10.3389/fmolb.2021.690899 (PMC8662366; doi:10.3389/fmolb.2021.690899)
Supplement: Supplementary file 1 [file DataSheet1.pdf]

Organelle Shaping

SPG3A *ATL1*
SPG58 *KIF1C*
SPG72 *REEP2*
SPG4 *SPAST*
SPG12 *RTN2*
SPG17 *BSCL2*
SPG31 *REEP1*
SPG33 *ZFYVE27*
SPG20 *KIAA0610*
SPG46 *GBA2*
SPG57 *TFG*
SPG61 *ARL6IP1*
SPG67 *PGAP1*
SPG69 *RAB3GAP2*

Microtubule Dynamics

SPG30 *KIF1A*
SPG72 *REEP2*
SPG4 *SPAST*
SPG10 *KIF5A*
SPG31 *REEP1*

Signaling

SPG3A *ATL1*
SPG18/37 *ERLIN2*
SPG4 *SPAST*
SPG6 *NIPA1*
SPG42 *SLC33A1*
SPG20 *KIAA0610*
SPG39 *PNPLA6*

Endo-membrane Trafficking

SPG3A *ATL1*
SPG4 *SPAST*
SPG6 *NIPA1*
SPG8 *KIAA019*
SPG10 *KIF5A*
SPG80 *UBAP1*
SPG11 *KIAA1840*
SPG15 *ZFYVE26*
SPG20 *KIAA0610*
SPG21 *ACP33*
SPG47 *AP4B1*
SPG48 *AP5Z1*
SPG50 *AP4M1*
SPG51 *AP4E1*
SPG52 *AP4S1*
SPG53 *VPS37A*
SPG57 *TFG*
SPG59 *USP8*
SPG78 *ATP13A2*

Degradation Pathways

SPG18/37 *ERLIN2*
SPG80 *UBAP1*
SPG11 *KIAA1840*
SPG15 *ZFYVE26*
SPG23 *DSTYK*
SPG48 *AP5Z1*
SPG49 *TECPR2*
SPG59 *USP8*
SPG60 *WDR48*
SPG62 *ERLIN1*
SPG76 *CAPN1*
SPG79 *UCHL1*

Mitochondrial Function

SPG9 *ALDH18A1*
SPG7 *SPG7*
SPG13 *HSPD1*
SPG31 *REEP1*
SPG20 *KIAA0610*
SPG28 *DDHD1*
SPG43 *C19orf12*
SPG55 *C12orf65*
SPG74 *IBA57*
SPG77 *FARS*
SPG78 *ATP13A2*
SPG83* *HPDL*

Metabolism/ Hormones

Amino Acid Metabolism

SPG9 *ALDH18A1*

Nucleotide Metabolism

SPG63 *AMPD2*
SPG64 *ENTPD1*
SPG(45/65) *NT5C2*

RNA Related

SPG70 *MARS*
SPG71 *ZFR*

Hormones

SPG22 *SLC16A2*
SPG66 *ARSI*

Transport

SPG30 *KIF1A*
SPG58 *KIF1C*
SPG4 *SPAST*
SPG10 *KIF5A*
SPG67 *PGAP1*

Lipid Metabolism

SPG18/37 *ERLIN2*
SPG17 *BSCL2*
SPG42 *SLC33A1*
SPG73 *CYTEC*
SPG5 *CYP7B1*
SPG11 *KIAA1840*
SPG26 *B4GALNT1*
SPG28 *DDHD1*
SPG35 *FA2H*
SPG39 *PNPLA6*
SPG46 *GBA2*
SPG54 *DDHD2*
SPG56 *CYP2U1*
SPG62 *ERLIN1*
SPG81 *EPT1*
SPG82 *PCYT2*
No SPG *ABHD16A*

Myelination

SPG35 *FA2H*
SPG39 *PNPLA6*
SPG44 *GJC2/ CX47*
SPG46 *GBA2*
SPG75 *MAG*
SPG1 *L1CAM*
SPG2 *PLP1*

Development

SPG3A *ATL1*
SPG4 *SPAST*
SPG6 *NIPA1*
SPG8 *KIAA0196*
SPG10 *KIF5A*
SPG33 *ZFYVE27*
SPG20 *KIAA0610*
SPG59 *USP8*
SPG68 *FLRT1*
SPG1 *L1CAM*
SPG22 *SLC16A2*
